# Selective impairment of methylation maintenance is the major cause of DNA methylation reprogramming in the early embryo

**DOI:** 10.1186/1756-8935-8-1

**Published:** 2015-01-09

**Authors:** Julia Arand, Mark Wossidlo, Konstantin Lepikhov, Julian R Peat, Wolf Reik, Jörn Walter

**Affiliations:** University of Saarland, FR 8.3, Biological Sciences, Genetics/Epigenetics, Campus A2.4, 66123 Saarbrücken, Germany; Epigenetics Programme, The Babraham Institute, Cambridge, CB22 3AT UK; Wellcome Trust Sanger Institute, Hinxton, CB10 1SA UK; Departments of Pediatrics and Genetics, Stanford University School of Medicine, 265 Campus Drive, Stanford, CA 94305 USA; Departments of Genetics and Obstetrics & Gynaecology, Stanford University School of Medicine, Institute for Stem Cell Biology & Regenerative Medicine, 265 Campus Drive, Stanford, CA 94305 USA

**Keywords:** DNA methylation reprogramming, Pre-implantation development, DNA methylation pattern, Deep hairpin bisulfite sequencing

## Abstract

**Background:**

DNA methylomes are extensively reprogrammed during mouse pre-implantation and early germ cell development. The main feature of this reprogramming is a genome-wide decrease in 5-methylcytosine (5mC). Standard high-resolution single-stranded bisulfite sequencing techniques do not allow discrimination of the underlying passive (replication-dependent) or active enzymatic mechanisms of 5mC loss. We approached this problem by generating high-resolution deep hairpin bisulfite sequencing (DHBS) maps, allowing us to follow the patterns of symmetric DNA methylation at CpGs dyads on both DNA strands over single replications.

**Results:**

We compared DHBS maps of repetitive elements in the developing zygote, the early embryo, and primordial germ cells (PGCs) at defined stages of development. In the zygote, we observed distinct effects in paternal and maternal chromosomes. A significant loss of paternal DNA methylation was linked to replication and to an increase in continuous and dispersed hemimethylated CpG dyad patterns. Overall methylation levels at maternal copies remained largely unchanged, but showed an increased level of dispersed hemi-methylated CpG dyads. After the first cell cycle, the combined DHBS patterns of paternal and maternal chromosomes remained unchanged over the next three cell divisions. By contrast, in PGCs the DNA demethylation process was continuous, as seen by a consistent decrease in fully methylated CpG dyads over consecutive cell divisions.

**Conclusions:**

The main driver of DNA demethylation in germ cells and in the zygote is partial impairment of maintenance of symmetric DNA methylation at CpG dyads. In the embryo, this passive demethylation is restricted to the first cell division, whereas it continues over several cell divisions in germ cells. The dispersed patterns of CpG dyads in the early-cleavage embryo suggest a continuous partial (and to a low extent active) loss of methylation apparently compensated for by selective *de novo* methylation. We conclude that a combination of passive and active demethylation events counteracted by *de novo* methylation are involved in the distinct reprogramming dynamics of DNA methylomes in the zygote, the early embryo, and PGCs.

**Electronic supplementary material:**

The online version of this article (doi:10.1186/1756-8935-8-1) contains supplementary material, which is available to authorized users.

## Background

The life cycle of mammals is characterized by two phases of major epigenetic reprogramming: first during migration of the primordial germ cells (PGCs) to the genital ridge in the developing embryo, and the second after fertilization during pre-implantation development [1]. These phases of epigenetic reprogramming involve changes in histone modifications and the activation of pluripotency-associated factors. Most intriguing is the accompanying reprogramming of DNA methylation, mainly characterized by a genome-wide decrease in DNA methylation [2–9]. The molecular control mechanisms for both genome-wide DNA demethylation processes remain unclear. In principle, demethylation of 5-methylcytosine (5mC) can be accomplished via an active enzymatic process or a passive replication-dependent process. Active DNA demethylation involves enzymes that remove either the methyl group or the whole base, accompanied by activation of ubiquitous DNA repair pathways [10].

In PGCs, a large proportion of DNA demethylation appears to occur by replication-associated passive demethylation, most likely influenced by 5-hydroxymethylcytosine (5hmC) [5, 11, 12]. However, mechanisms of active DNA demethylation by enzymatic conversion of 5mC (or 5hmC) are also likely to contribute. Thus, mechanisms involving the deamination of 5mC to thymine by activation-induced deaminase (AID) or other non-deamination dependent repair pathways have been suggested [2, 13].

In the zygote, a substantial loss of 5mC in the paternal pronucleus before replication has been shown by immunofluorescence (IF) analyses. For a long time, this was interpreted to represent genome-wide active loss of DNA methylation before replication [14–16]. An observed major drop in DNA methylation by bisulfite sequencing after replication [16] indicated that 5mC is not immediately replaced with unmodified cytosine but rather converted into a different chemical status. Indeed with the discovery of 5hmC, it became clear that the conversion of 5mC into 5hmC and other oxidized forms catalyzed by Tet3 are likely mechanisms to initiate the progressive loss of 5mC [17–19]. Hence, the idea of an active paternal genome demethylation had to be reconsidered. In addition, as 5hmC appears to be diluted during further cleavage stages, this modification is likely to be the major cause of DNA demethylation during the cleavage stages, caused by a continued impairment of maintenance methylation function of the DNA methyltransferase Dnmt1 [20]. However, even high-resolution IF analyses left open the question of how double-stranded DNA methylation patterns are affected in the first rounds of cell divisions. Recent studies using enrichment-based profiling (reduced representation bisulfite sequencing; RRBS) and genome-wide bisulfite sequencing provided evidence that the most dramatic effect of DNA demethylation takes place at the zygotic stage [6, 9]. However, although these analyses revealed an overall dilution effect on DNA methylation, they did not address how DNA methylation patterns on both complementary DNA strands (complementary CpG dinucleotide; CpG dyads) are affected, and therefore could not draw conclusions on the possible mechanisms controlling DNA methylation.

In this study, we address these open questions by simultaneously analyzing the changes in DNA methylation patterns on both complementary DNA strands during the early phases of mouse development. We used hairpin bisulfite sequencing [21, 22] to investigate the replication-dependent DNA methylation pattern dynamics at specific repetitive elements such as the L1Md_Tf (hereafter referred to as L1), major satellites (mSat) and IAPLTR1 (IAP). L1 and mSat were chosen because they have previously been shown to undergo DNA demethylation, whereas IAPs were reported to be resistant to DNA demethylation in the zygote [9, 16, 23, 24]. Our analysis follows the fate of DNA methylation at these elements, starting from mouse germ cells over the first cleavage stages up to the blastocyst stage and PGCs. In addition, zygotes and two-cell embryos were analyzed at precisely timed stages during the cell cycle in order to discriminate between pre-replicative and post-replicative states. Additionally, we separately isolated maternal and paternal zygotic pronuclei for our analyses in order to address differences previously reported for both sets of chromosomes [6, 14, 25]. Our comparative analysis provides clear evidence for the presence of a DNA methylation maintenance function in early embryos, which is partially impaired during the first cell cycle. We also found continuous presence of hemimethylated CpG dyads across the first cell divisions. Our findings suggest a complex interplay between possible mechanisms of DNA methylation reprogramming (that is, demethylation and *de novo* methylation) and DNA methylation maintenance in the early mouse embryo.

## Results

### DNA methylation reprogramming of L1, mSat, and IAP in the zygote is characterized by an increasing amount of hemimethylated CpG dyads

**Figure 1:**
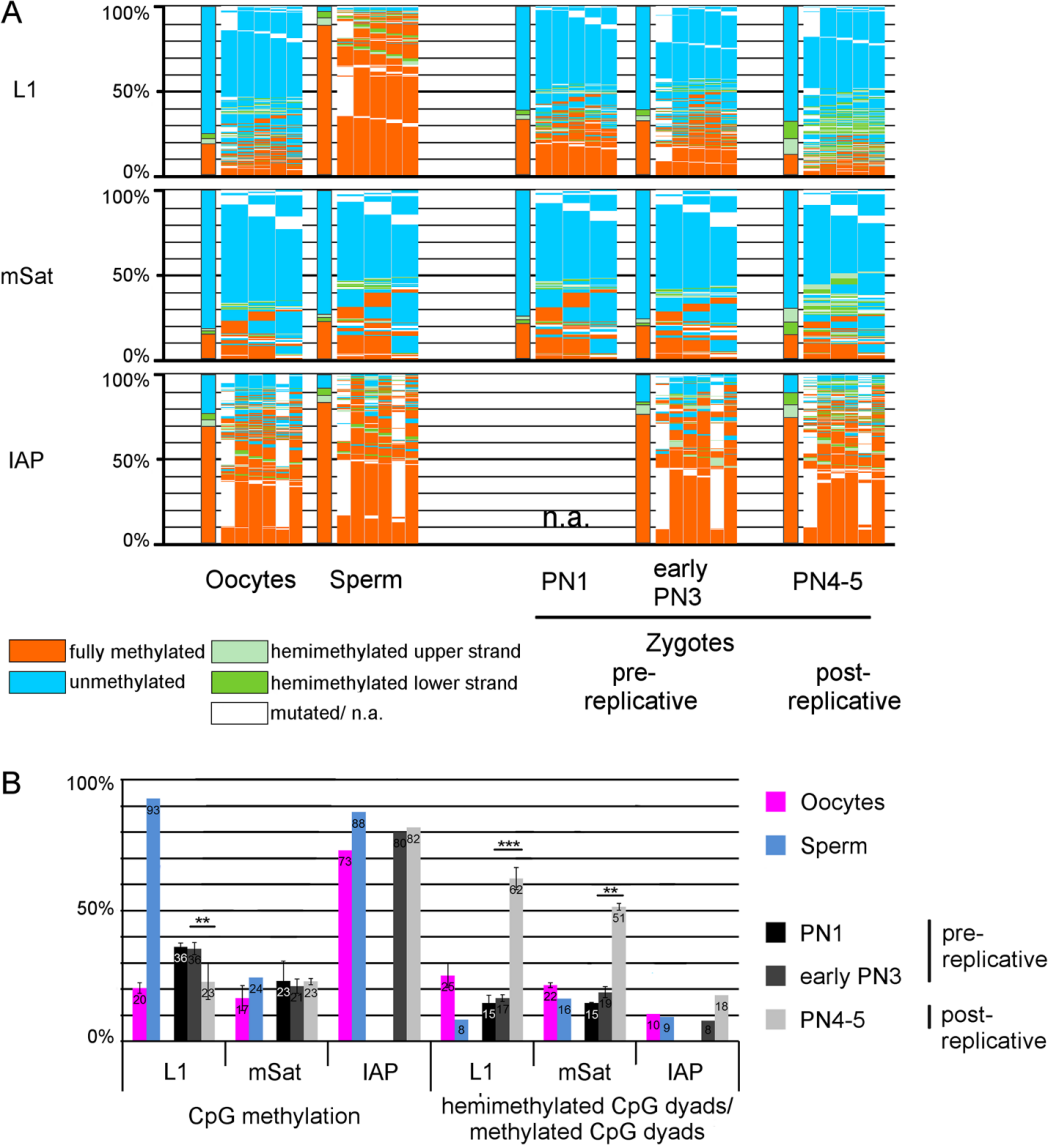
**DNA Methylation patterns of L1Md_Tf (L1), major satellites (mSat), and IAPLTR1 (IAP) in germ cells and three pronuclear stages (PN) of mouse zygotes. (A)** DNA methylation patterns, Bars are the sum of the DNA methylation status of all CpG dyads. The map next to the bar represents the distribution of methylated sites. Each column shows neighbored CpG dyads, and each line represents one sequence read. The reads in the map are sorted first by fully methylated sites and then by hemimethylated CpG dyads. Red, fully methylated CpG dyads; light green and dark green, hemimethylated CpG dyads on the upper and lower strand; blue, unmethylated CpG dyads; white, mutated or not analyzable. As 5-methylcytosine (5mC) and 5-hydroxymethylcytosine (5hmC) cannot be discriminated by bisulfite sequencing, "mC" should be considered to mean 5mC or 5hmC throughout the paper, and equally "C" (cytosine) should be considered to mean C, 5-formylcytosine (5fC), or 5-carboxycytosine (5caC). **(B)** Absolute DNA methylation level and percentage of hemimethylated CpG dyads in relation to all methylated CpG dyads. DNA Methylation patterns of L1, mSat and IAP were analyzed in germ cells (oocytes and sperm) and different PN stages (PN1 and early PN3 are before replication, PN4 to PN5 are after replication) using deep hairpin bisulfite sequencing (DHBS). DNA methylation pattern changes can be observed following the first DNA replication after fertilization; in all elements an increasing amount of hemimethylated CpG dyads can be seen, and for L1 DNA demethylation can also be observed.

To precisely determine DNA methylation symmetry of individual CpG dyads at single nucleotide resolution, we performed deep hairpin bisulfite sequencing (DHBS). We first determined the ground state of methylation at L1, IAP, and mSat in mature germ cells (sperm and oocytes). In line with previous data, we found that mSat are hypomethylated and IAP hypermethylated in both oocytes and sperm, whereas L1 elements are hypermethylated in sperm and hypomethylated in oocytes (Figure 1) [9, 23, 24]. It should be note that bisulfite sequencing cannot distinguish 5mC from 5hmC or unmodified C from 5-formylcytosine (5fC) or 5-carboxycytosine (5caC), therefore the term "methylated DNA sequences" refers hereafter to the sum of 5mC and 5hmC, and accordingly, hemimethylated CpG dyads are the sum of hemi-5mC and hemi-5hmC. DHBS showed that the methylated dyads of both sperm and oocyte chromosomes contain a high level of fully methylated CpG dyads (Figure 1). In early pre-replicative zygotes, DNA methylation represents the average of oocyte and sperm values, with the number of fully methylated CpG dyads initially remaining unaltered (Figure 1). At post-replicative pronuclear stages of the zygote (for examples of staging see Additional file 1), DNA methylation patterns change dramatically, with an extensive loss of fully methylated CpG dyads, accompanied by a strong increase in hemimethylated CpG dyads. After replication, more than 50% of all methylated CpG dyads of L1 and mSat copies are in a hemimethylated state. By contrast, at IAP, almost all methylated CpG dyads remain fully methylated. Hence, despite an overall dramatic shift towards hemimethylated CpG dyads, a substantial proportion of fully methylated CpG dyads remain in certain L1 and mSat copies and in almost all IAP. This finding argues for a selective control of maintenance methylation during the first zygotic replication.

### DNA methylation patterns change differently in maternal and paternal pronuclei

**Figure 2:**
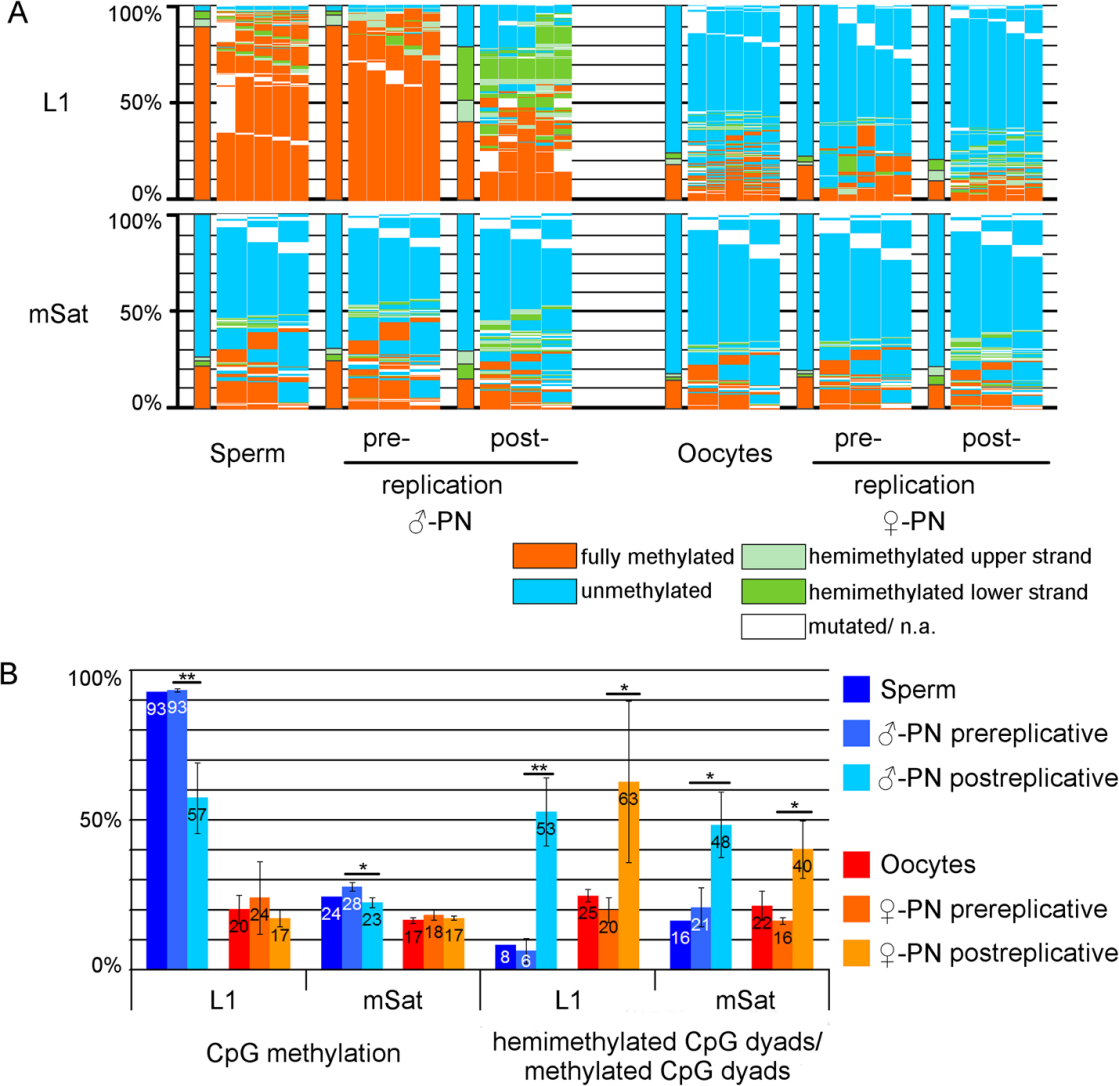
**DNA methylation patterns of major satellites (mSat) and L1Md_Tf (L1) of separated pronuclei of pre- and post-replicative stages of mouse zygotes and germ cells. (A)** DNA methylation patterns; for explanation, see Figure 1, **(B)** Absolute DNA methylation level and percentage of hemimethylated CpG dyads in relation to all methylated CpG dyads. Paternal and maternal pronuclei were separated by micromanipulation before and after DNA replication, and L1 and mSat were analyzed with deep hairpin bisulfite sequencing (DHBS). As comparison, we added DHBS data from germ cells. Only after replication was a decrease in DNA methylation found on paternal chromosomes; accompanied by an increase in hemimethylated CpG dyads on both maternal and paternal chromosomes.

IF analyses of zygotes with antibodies against 5mC and 5hmC strongly suggested that the conversion of 5mC to 5hmC is particularly pronounced in the paternal DNA [17–19], and may serve as a signal for DNA demethylation. Using single nucleotide polymorphisms (SNPs) in single copy genes, we and others have found a more pronounced zygotic demethylation in paternal copies [6, 9, 25]. To investigate this on a broader genomic scale, we performed DHBS and conventional single-stranded bisulfite sequencing on DNA from isolated paternal and maternal pronuclei, and compared the methylation patterns to those of the corresponding germ cells (Figure 2; see Additional file 2). The analysis confirmed a strong and significant reduction in DNA methylation in paternal L1 elements, whereas the reduction in mSat was comparatively small (with low significance) (Figure 2). The demethylation of paternal L1 copies was associated with an increase in the ratio of hemimethylated CpG dyads (Figure 2b). Interestingly, the overall level of L1 and mSat methylation in maternal pronuclei remained largely unchanged, whereas the composition of DNA methylation patterns showed a strong increase in hemimethylated CpG dyads relative to all methylated dyads. We have recently shown that the amount of 5hmC also increases in the maternal pronuclei, whereas the level of 5mC decreases [19]. The increased levels of hemimethylated dyads could be linked to this effect.

### Major DNA methylation changes in the zygote require DNA synthesis and replication

**Figure 3:**
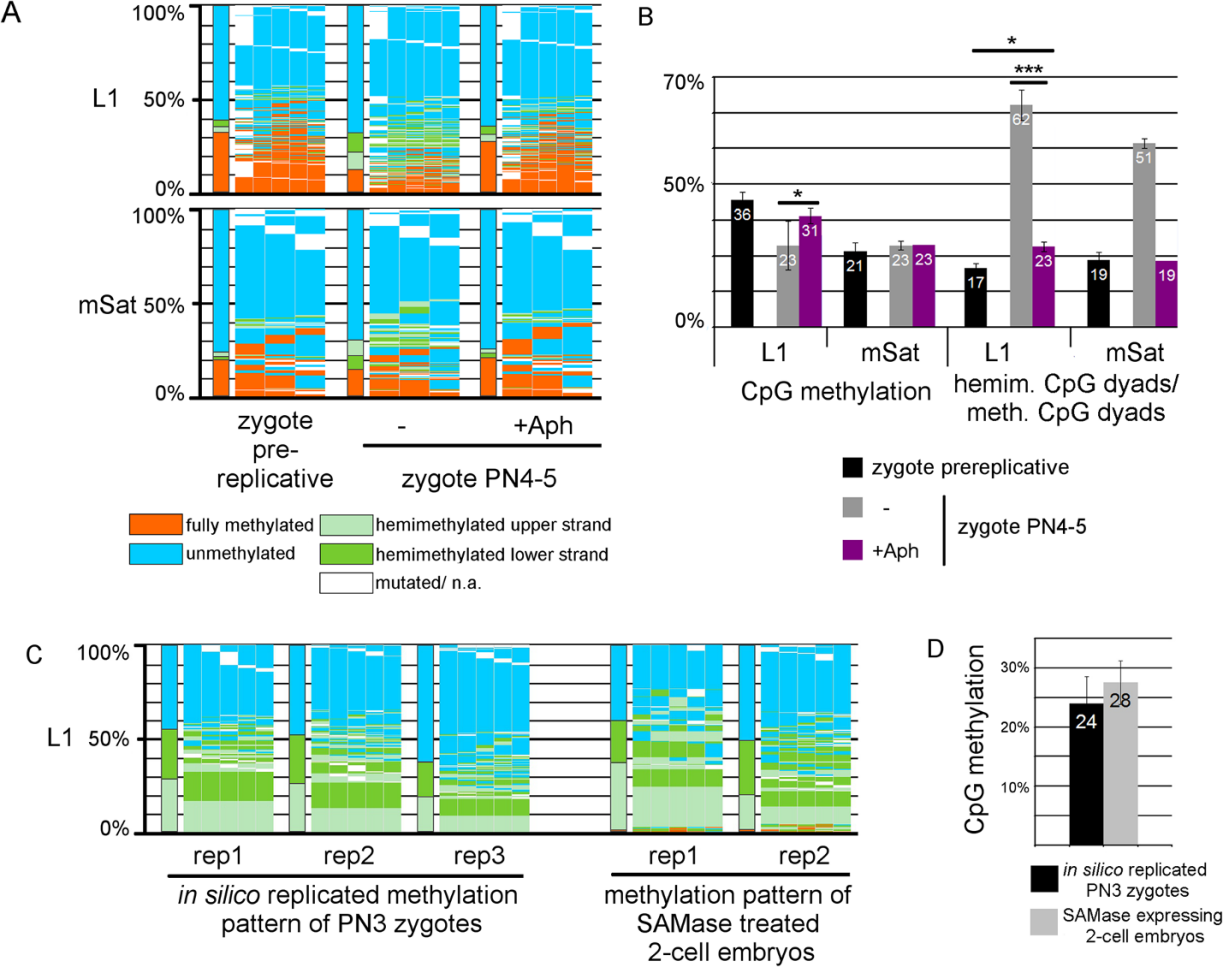
**Replication dependency of DNA methylation reprogramming in the zygote. (A)** DNA methylation patterns (for explanation see Figure 1) and **(B)** absolute DNA methylation level and percentage of hemimethylated CpG dyads in relation to all methylated CpG dyads of L1 and mSat in +/- aphidicolin-treated (+/- replication-blocked) PN4-5 zygotes. Aphidicolin treatment leads to diminished DNA demethylation. **(C,D)** DNA methylation patterns of replicates (rep) and **(C)** absolute DNA methylation level of SAMase-treated early (pre-replicative) two-cell embryos. SAMase diminishes all methylation events that are dependent on S-adenosyl-methionine (SAM). The patterns were compared with those of *in silico* replicated zygotes with no DNA methylation maintenance (see Methods). The DNA methylation profiles of the biological replication without methylation events (SAMase-treated two-cell embryos) are very similar to those events simulated *in silico* without methylation.

To better understand the connection between DNA methylation and DNA *de novo* synthesis, we analyzed zygotes treated with aphidicolin. Aphidicolin blocks all DNA synthesis but it does not affect zygotic pronuclear maturation (see Additional file 3). We found that DHBS methylation patterns of mSat copies in late-stage aphidicolin-treated zygotes remained unchanged compared with those of pre-replicative zygotes (Figure 3a,b). In L1 copies, we found a very small decrease in DNA methylation, visible as altered composition of hemimethylated and fully methylated sequences (Figure 3b). We conclude that the observed major changes in DNA methylation (5mC and 5hmC) in the zygote are mainly dependent on DNA synthesis (DNA repair and DNA replication).

Next, we investigated the effect of blocking all DNA methylation reactions (*de novo* and maintenance) during the first cell cycle, with the goal of detecting changes in DNA methylation patterns that are attributable to active DNA demethylation but are replication associated. To achieve this, we injected mRNA of the SAMase gene into early, pre-replicative zygotes. SAMase is a T3 bacteriophage-specific enzyme, which degrades S-adenosyl-methionine (SAM) [26]. The depletion of endogenous SAM pool by SAMase blocks all methylation reactions in which SAM serves as a methyl group donor. Expression of SAMase in zygotes led to visible reduction of 5mC when the resulting two-cell embryos were analyzed by IF (see Additional file 4). In paternal pronuclei of post-replicative zygotes, the perinucleolar rings, mainly enriched with mSat repeats, are usually positively stained by anti-5mC antibody, but when SAMase was expressed, the IF signals at perinucleolar rings were strongly reduced (see Additional file 4).

The DHBS methylation patterns obtained from the SAMase-treated post-replicative stages showed almost complete lack of fully methylated CpG dyads (Figure 3c), validating the inhibition of *de novo* and maintenance methylation. To identify events attributable to active DNA demethylation, we compared these DNA methylation patterns with those obtained by simulated replication without any maintenance or *de novo* methylation events of pre-replicative zygotes (*in silico* replication; see Methods). Neither the methylation patterns nor the levels showed significant differences (Figure 3c,d), indicating that mainly passive DNA demethylation events occur during the first replication in the zygote.

Taken together, both experiments clearly show that DNA synthesis and replication is necessary for substantial DNA demethylation. In addition, they suggest that partially impaired maintenance methylation is likely to be the major cause of 5mC/5hmC demethylation in the paternal chromosomes during the first cell cycle.

We note that our data do not exclude a (minor) contribution by active DNA demethylation mechanisms. Two observations support a minor contribution of active DNA demethylation: First, we found that in aphidicolin-treated zygotes, there was a small but recognizable change in DHBS patterns, when early zygotes were compared against late aphidicolin-treated zygotes, suggesting a small replication-independent change in the 5mC/5hmC content (for example increase in hemimethylated CpG dyads in L1) (Figure 3), in line with our previous observations [16]. Second, the strong increase in unmethylated CpG positions in paternal pronuclei DNA cannot be explained by a selective passive dilution mechanism alone (Figure 2). The contribution of oxidized forms of 5mC (5fC, 5caC) to the observed increase in "unmethylated" cytosines remains unclear because the standard bisulfite technology does not discriminate unmodified cytosine and 5fC or 5caC. It is likely that some of the methylation changes are influenced by pre-replicative and post-replicative oxidation of 5hmC into 5fC or 5caC, which would not be detected by bisulfite sequencing. These findings indicate a minor contribution of active demethylation with the conversion of 5mC to unmodified cytosines.

### Mosaic DNA methylation during the first cleavage stages suggests a constant loss and gain of methylation

**Figure 4:**
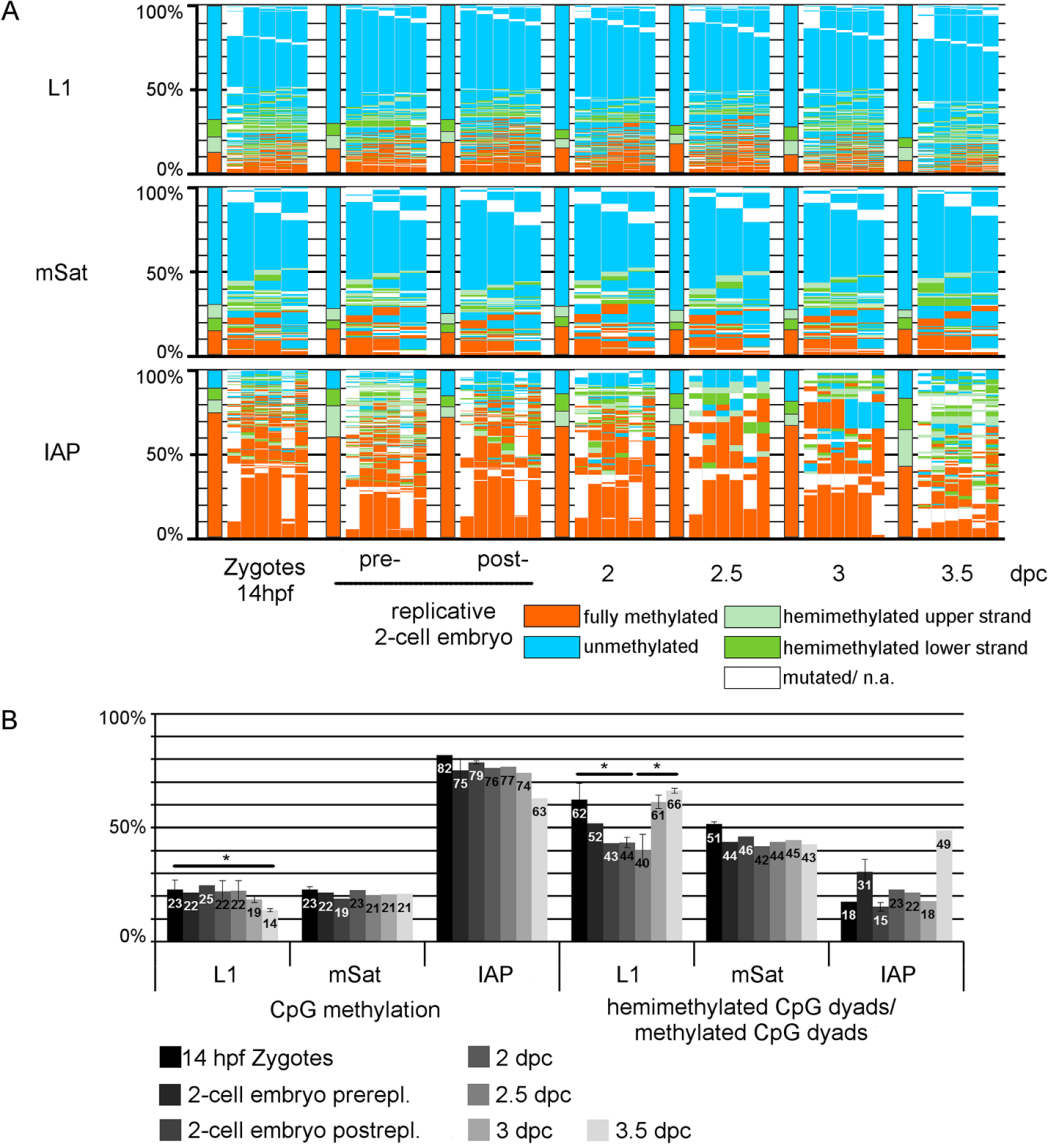
**DNA methylation pattern of L1Md_Tf (L1), major satellites (mSat), and IAPLTR1 (IAP) in cleavage stage mouse embryos. (A)** DNA methylation patterns (for explanation see Figure 1) **(B)** Absolute DNA methylation levels and percentage of hemimethylated CpG dyads in relation to all methylated CpG dyads. Two-cell embryos were analyzed before and after replication, and further-cleavage stage embryos were analyzed at 12-hour intervals to determine methylation changes during replications. DNA methylation pattern remained stable until the morula stage, when a further drop in methylation occurred at L1 and IAP. Interestingly, over the course of the cleavage stages the amount of hemimethylated CpG positions remained equally high.

Having determined the baseline state of DNA methylation at the end of the first cell cycle, we then followed the DNA methylation patterns during the first cleavage stages. Previous work has suggested continuous passive DNA demethylation and/or non-maintenance of 5hmC [1, 20], leading to further dilution of DNA methylation. However, when we followed the methylation pattern of two-cell embryos before and after replication, we did not observe a significant decrease in DNA methylation during the second DNA replication event (Figure 4). In fact, in IAP elements we actually found an increase in fully methylated CpG dyads at this point.

Next, we analyzed the DNA methylation in embryos collected at 12-hour intervals (Figure 4, Additional file 5). Until the early morula (8 to 16-cell stage, 2.5 days post-coitum (dpc)) the overall methylation level and proportional distribution of all elements remained largely constant, accompanied by consistently high proportions of hemimethylated CpG dyads relative to all methylated CpG dyads. Only at the transition between late morula (16 to 32 cells, 3 dpc) and blastocyst stage (>64 cells, 3.5 dpc), we observed a further decrease in DNA methylation at L1 and IAP elements, along with an increase in hemimethylated CpG dyads (Figure 4).

The maintenance of a high proportion of dispersed hemimethylated positions in maternal sequences in the zygote and between the two-cell and morula stages suggests that methylation maintenance involves a "balanced" loss and gain of methylation over several rounds of replication. A persistence of dispersed methylation profiles at CpG dyads can be caused by constantly maintaining high levels of highly oxidized forms (not detectable by bisulfite sequencing) and/or by a loss (impaired maintenance) and re-gain of 5mC by *de novo* methylation. As Tet3 is known to be absent from the two-cell stage onwards, the constant *de novo* methylation scenario is more likely to occur.

### DNA is progressively demethylated in replicating PGCs

**Figure 5:**
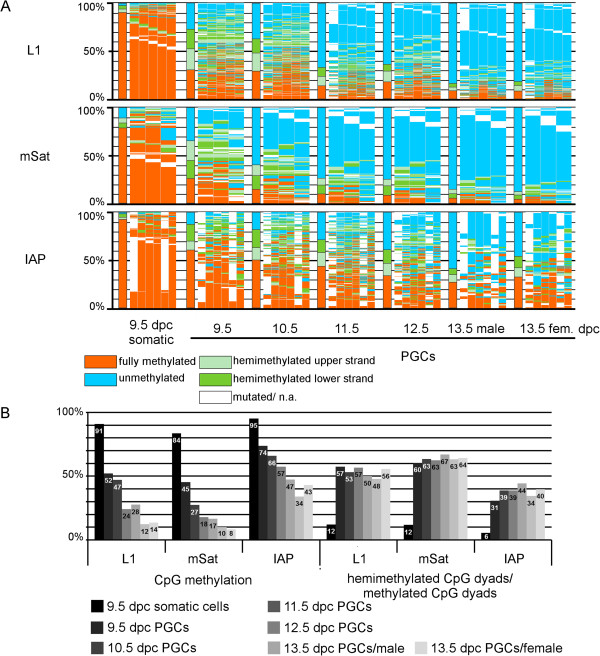
**DNA Methylation pattern of L1Md_Tf (L1), major satellites (mSat), and IAPLTR1 (IAP) in primordial germ cells (PGCs). (A)** DNA methylation patterns (for explanation see Figure 1). **(B)** Absolute DNA methylation levels and percentage of hemimethylated CpG dyads in relation to all methylated CpG dyads. PGCs were sorted and analyzed at 24-hour intervals from 9.5 until 13.5 days post-coitum (dpc). DNA methylation decreased continuously, with a stable relative level of hemimethylated CpG positions.

Low coverage and genome-wide data suggested a stepwise decrease in DNA methylation during PGC development [4, 5, 27, 28]. To systematically investigate if this stepwise loss of DNA demethylation in PGCs is linked to replication-dependent accumulation of hemimethylated CpG dyads, we performed DHBS for mSat, IAP, and L1 in staged PGCs and somatic cells between 9.5 dpc and 13.5 dpc (see also [5] for L1). In early PGCs (9.5 dpc) DNA methylation was already reduced compared with surrounding somatic cells (Figure 5). Somatic cells showed a very low level of hemimethylated CpG dyads, whereas 50% of all methylated CpG dyads in 9.5 dpc PGCs exhibited a hemimethylated state, indicating that the DNA demethylation process is already in progress in 9.5 dpc PGCs. Between 9.5 and 13.5 dpc, we observed continuous progression of DNA demethylation, accompanied by a persistently high proportion of hemimethylated CpG dyads.

Hence, as in early embryos, we found a strong correlation between overall loss of DNA methylation and the presence of hemimethylated CpG dyads. This strongly argues for a continuous selective impairment of maintenance methylation as a major mechanism of demethylation in PGCs (see also [5]). In contrast to early-cleavage embryos, this process appears to occur continuously in PGCs over several replication cycles, and is apparently not accompanied by *de novo* methylation.

## Discussion

In our study, we analyzed the fate of symmetrical DNA methylation across the first cell divisions in the mouse pre-implantation embryo and in PGC development. These developmental periods are characterized by an extensive reprogramming of genome-wide DNA methylation patterns, mainly extensive erasure of 5mC. We used DHBS to precisely follow the dynamics of DNA methylation patterns in single DNA strands of cells isolated at defined stages of these reprogramming phases. This staged DHBS profiling allowed us to draw mechanistic interpretations from the fate of methylation on single DNA molecules. The analyzed repetitive elements represent widely dispersed different reprogramming classes resistant or sensitive to demethylation, which, as we recently reported, also recapitulated pattern formation at single gene loci in embryonic stem cells (ESCs) [21].

**Figure 6:**
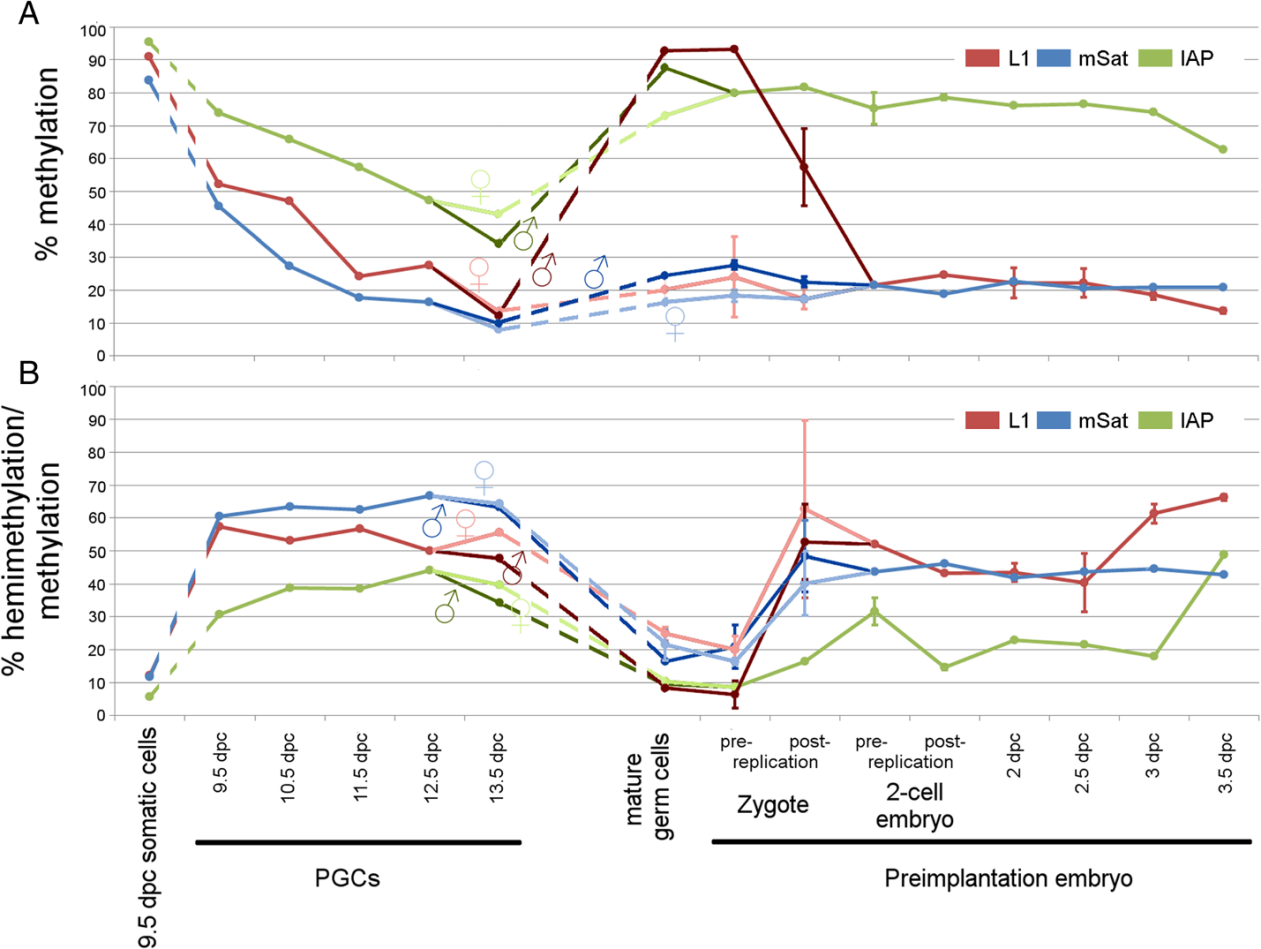
**Summary of DNA methylation changes in primordial germ cells (PGCs) and pre-implantation embryos. (A)** Absolute DNA methylation levels and **(B)** percentage of hemimethylated CpG dyads in relation to all methylated CpG dydas (red, L1Md_Tf (L1); blue, major satellites (mSat); green, IAPLTR1 (IAP)). Note that from 13.5 dpc PGCs to the two-cell stage the values are depicted separately for maternal and paternal chromosomes, respectively, and thereafter depicted as combined values.

One major observation of our study is that DNA demethylation is mainly caused by partial impairment of DNA methylation maintenance during replication. This can be deduced from the significant increase in hemimethylated CpG dyads in both pre-implantation embryos and during PGC development. The second observation is that this process appears to be continuous during PGC development, but discontinuous in the developing early embryo. In the early embryo, a decrease in methylation occurred at two developmental points: in the zygote (mainly reducing the level of paternal methylation) and around 3 dpc (that is, around the 32-cell stage). Between the two-cell stage and day 3 of development (mainly up to the 16-cell stage) the chromosomes maintained a largely constant level of methylation. At L1 and mSat elements, we observed the presence of dispersed hemimethylated CpG dyads. Such "noisy" patterns were maintained up to day 3 of embryonic development at a constant level (Figure 6). Similar noisy patterns are found in ESCs lacking Dnmt1 [21], or in ESCs cultured in 2i medium [29]. Shipony *et al*. also recently reported "noisy" CpG methylation for certain DNA regions of ES cell clones [30].

During the revision of our manuscript, two other groups published RRBS studies showing that passive demethylation is the main cause of DNA demethylation in the zygote [31, 32]. Both groups also reported small but significant demethylation of maternal chromosomes in the zygote; however, their analysis was not sufficiently deep to detect that the demethylation of L1 and mSat sequences is counteracted and "balanced" by *de novo* methylation. They also did not follow this across subsequent cell divisions where we found this process to be maintained. We therefore postulate that *de novo* methylation (most likely by Dnmt3a) accompanies the reprogramming events in the early embryo as previously suggested [25]. In addition, the persistence of symmetrically methylated CpG dyads in IAP elements during the early-cleavage stages strongly suggests that the machinery for strict methylation maintenance must be present in the early embryo. In conclusion, our analysis provides a differentiated picture of the various mechanisms involved in shaping of a specific DNA methylation program following fertilization. We speculate the persistence of "noisy" patterns may be important for developmental potency and lineage decisions in the early embryo.

The molecular mechanisms responsible for selective impairment of maintenance methylation during the first cell cycle are still unclear. The conversion of 5mC to 5hmC, 5fC, and 5caC may play a crucial role. Reprogramming of DNA methylation in the zygote can be initiated by the oxidation of 5mC by Tet3 [17, 19]. Indeed, DNA demethylation of L1 was shown to be dependent on Tet3 activity [17], and *in vitro* data suggest that Dnmt1 fails to maintain methylation at CpG containing hydroxymethylated cytosines [33]. Furthermore, RRBS analysis of Tet3 KO zygotes suggests that replication-dependent demethylation is partly dependent on oxidation of 5mC by Tet3 [31, 32]. The targeted avoidance of passive DNA demethylation can accordingly be executed via interaction with specific factors, such as Stella, which impairs oxidation by Tet3 [19, 34]. Previous findings corroborate this assumption: the accumulation of hemimethylated CpG dyads in both pronuclei corresponds well with increase in 5hmC, as 5hmC is also detected in maternal pronuclei at later stages [9, 19]. Furthermore, another study published during the proofs of this manuscript suggests that there might be Tet3-dependent and other mechanisms-dependent demethylation pathways which are redundant [35].

In addition to a clear predominance of apparently passive demethylation mechanisms in the mouse zygote, careful inspection of methylation patterns identified a lower prevalence of active DNA demethylation, in line with previous and two very recent reports [16, 24, 25, 31, 32]. Hence, a moderate and sequence-specific contribution of active mechanisms to DNA demethylation is apparently contributing to reprogramming. However, because the bisulfite reaction does not discriminate between unmodified cytosine, 5fC, and 5caC [36, 37], it is unclear whether our data indicate formation of unmodified cytosines by an active, probably repair-coupled, process, or if the changes in patterns simply reflect the generation of higher oxidized forms of 5mC. Indeed, reports indicate the presence of 5fC/5caC in the zygote using IF analysis [38] and a specifically modified bisulfite sequencing protocol [9]. However, a recent study found no or only very little detectable 5fC/5caC at specific actively demethylated sequences in zygotes, and suggested that they are further processed by pathways such as base excision repair to yield unmodified cytosine [31].

High-resolution IF analysis in a previous study suggested a replication-dependent dilution of 5hmC during further cleavage stages [20]. However, this scenario does not correspond to our molecular findings beyond the first cleavage stage. The model of a cell division (replication)-dependent dilution of modified cytosines (5hmC or 5mC) would predict a further decrease of bisulfite treatment-resistant cytosines. From the two-cell embryo stage up to the early morula stage, the overall methylation patterns remained remarkably stable, maintaining a constant amount of hemimethylated CpG dyads (Figure 4). The finding that a great proportion of CpG dyads retained a fully methylated state after continuous replication cycles indicates that maintenance methylation is not completely absent during the first cleavage stages, but that it is apparently impaired at selected sequences. These findings can be explained by the following scenario. The impairment of maintenance methylation by 5hmC is highest during the first cell cycle at selected sequences. In the absence of Tet3 and other factors at later stages [19], DNA methylation maintenance is impaired to a lesser extent and/or further passive loss is counteracted by enhanced *de novo* methylation carried out by *de novo* methyltransferases, probably by Dnmt3a, which has been shown to be present in the zygote and later stages [17]. By contrast, Dnmt3a and Dnmt3b are not expressed in PGCs ( [39] and own unpublished observations), where DNA methylation continuously decreases during subsequent cell divisions (Figure 5).

This work underlines the need to more closely characterize the various contributions of DNA methyltransferase for DNA methylation persistence and their temporal control during early embryogenesis, in order to gain a better understanding of DNA methylation reprogramming processes.

## Conclusions

Using DHBS, we have generated the first deep resolution methylation maps of CpG dyads of specific repetitive element classes across individual DNA replications and cell divisions in the early mouse embryo and during PGC formation (summarized in Figure 6). In PGCs, CpG methylation continuously decreases across consecutive cell divisions. This process is clearly linked to an accumulation of hemimethylated CpG dyads, reflecting a replication-dependent "passive" demethylation process. In the early embryo, such a process is confined to the paternal chromosomes, and occurs only during the first zygotic DNA replication. In the following cleavages and on maternal chromosomes in the late zygote, there is no loss of methylation but rather the maintenance of a constant degree of hemimethylated CpG dyad patterns at various repetitive elements. Our data suggest that in the embryo, incomplete passive and to a much lesser extent active demethylation mechanisms are antagonized by partial (*de novo*) methylation mechanisms to precisely maintain a development specific level of DNA methylation. Oxidation of 5mC by Tet enzymes is probably involved in the balance of these antagonistic enzymatic activities. In conclusion, during both major reprogramming phases in development, there is a rather dynamic DNA methylation landscape instead of a simple copying mechanism of the methylation pattern as seen in somatic cells. The establishment of these highly dynamic DNA methylation patterns is likely to be an important step in the generation of a totipotent and pluripotent epigenome and subsequent cell fate decisions in early embryogenesis.

## Methods

All animal experiments were carried out according to German Animal Welfare law in agreement with the authorizing committee.

### *In vitro*fertilization of mouse oocytes and manipulation of zygotic development

For *in vitro* fertilization (IVF), sperm was isolated from the cauda epididymis of adult (C57BL/6 × CBA) F1 male mice, and pre-incubated for 1.5 h in modified Embryomax KSOM Embryo culture medium (Merck Millipore, Darmstadt, Germany) (3 mg BSA/ml and 5.56 mM glucose in KSOM) supplemented with 27 mg BSA/ml. Mature oocytes from superovulated (C57BL/6 × CBA) F1 female mice were collected 14 h post-human chorionic gonadropin (hCG) injection according to the standard procedures [40]. Cumulus–oocyte complexes and capacitated sperm were placed into a 400 μl drop of modified KSOM medium (see above) at 37°C in a humidified atmosphere of 5% CO_2_ and 95% air. For the treatment with aphidicolin, 3 μg/ml aphidicolin was added at 4 hours post-fertilization (hpf). For collection of different PN stages, IVF-derived zygotes were stained with 5 μg/ml Hoechst 33342 for 30 min before the desired time points and correct PN staging, and contamination with sperm or cumulus cells was monitored by Hoechst staining and embryo by embryo selection under a fluorescent microscope. The classification of PN stages was performed as described previously [15, 16, 41], with the pronuclear morphology and hpf taken into consideration.

### Collecting embryos from natural breeding

Superovulated (C57BL/6 × CBA) F1 female mice were mated with (C57BL/6 × CBA) F1 male mice. At embryonic day (E)1.5, two-cell embryos were flushed from the oviduct and incubated further in M16 (Sigma-Aldrich, St Louis, MO, USA). Embryos were collected at 12-hour intervals starting from 2 dpc (2 dpc: late 4-cell stage/early 8-cell stage; 2.5 dpc: late 8-cell stage/16 cell stage; 3 dpc: morula stage) until blastocyst stage at 3.5 dpc (see Additional file 5).

### Pronuclei isolation

IVF-derived zygotes at 7 or 13.5 hpf were incubated with 5 μg/ml cytochalasin B, 2 μg/ml nocodazole, and 5 μg/ml Hoechst 33342 for 30 min in KSOM. Following this, the maternal and paternal pronuclei were separated using a micromanipulator under a Zeiss AxioVert 200 M inverted microscope (Zeiss, Germany) in M2 medium without BSA supplemented with 1% Polyvinylpyrrolidone (PVP), 5 μg/ml cytochalasin B, and 2 μg/ml nocodazole. The parental origin of the pronuclei was determined by the size of the pronuclei and their location in relation to the polar body using Hoechst 33342 staining. Only clearly classifiable pronuclei were collected.

### SAMase expression and injection into zygotes

The T3 bacteriophage SAMase coding sequence was amplified by PCR from T3 bacteriophage DNA, and inserted into a pET28b0-based vector, containing an enhanced green fluorescent protein (eGFP) coding sequence, followed by the 3′ untranslated region sequence of the mouse *TRF2* gene and downstream poly(83A) sequence (adopted from the pcDNA3.1EGFP-poly(A) plasmid, described in [42]). The resulting plasmid was used as template for *in vitro* transcription (MessageMax T7 ARCA-Capped Message transcription kit, Epicentre Biotechnologies, Madison, WI, USA) to produce mRNA, encoding for the SAMase-eGFP fusion protein. The mRNA was injected into early zygotes 2 to 4 hpf, and the injected zygotes were allowed to develop further for 16 hours until they reach late zygote or early two-cell stage (after first S-phase, before second S-phase). The translation efficiency was monitored by eGFP fluorescence.

### Isolation of PGCs

Genital ridges from Oct4-GFP transgenic embryos [43] were isolated from 9.5–13.5 dpc embryos then treated with trypsin, and single GFP-positive cells were collected manually using an inverted fluorescence microscope Zeiss AxioVert 200 M and micromanipulators (TransferMan NK2; Eppendorf, Germany). The sex of the embryos at 13.5 dpc was determined by the arrangement of the PGCs in the gonad. Each sample contained at least 40 PGCs. As a control, we collected GFP-negative cells from 9.5 dpc embryos.

### Hairpin bisulfite analysis

Embryos/pronuclei and a medium control from the last washing step were supplemented with 100 ng salmon sperm DNA and treated with proteinase K (0.2 mg/ml in 2 mM Tris–HCl, 1 mM EDTA), followed by hairpin bisulfite analysis as described previously [21] with the following changes. We analyzed 5 to 15 embryos/pronuclei and 40 to 50 PGCs per biological replica. For L1 analysis, the restriction enzyme *Bsa*WI was used (3 hours at 60°C) and for IAP analysis, the following primers and PCR conditions were used: forward TTTTTTTTTTAGGAGAGTTATATTT, reverse ATCACTCCCTAATTAACTACAAC, 45 cycles (95°C for 1 minute, 51°C for 1.5 minutes, 72°C for 1 minute). For L1 and mSat, the cycle number for the PCR was increased to 45 for L1 and 40 for mSat, respectively. Details of the results of the hairpin bisulfite sequencing of the different biological replicates and the number of replicates analyzed are given (see Additional file 6). Raw data can be obtained upon request.

### *In silico*replication

To mimic the situation of complete absence of DNA methylation maintenance (passive demethylation) during the first DNA replication in the zygote, we halved the methylation at all CpG dyads (pre-replicative state), while maintaining their relative neighborhood localization. Thus, unmethylated CpG dyads will give rise to two sequences with each having a completely unmethylated CpG dyad, hemimethylated CpG dyads will give rise to one sequence with a hemimethylated CpG dyad and the other with an unmethylated CpG dyad, and fully methylated CpG dyads will give rise to two sequences with hemimethylated CpG dyads.

## Electronic supplementary material

Additional file 1: **Representative images of Hoechst 33342-stained mouse zygotes.** Discrimination of developed zygotes was performed by hours and the morphology of the pronuclei (PN) as described previously [16]. PN1 and early PN3 represent pre-replicative PN stages and PN4 to PN5 the post-replicative PN stages. PB, polar body. (PNG 269 KB)

Additional file 2: **Comparison of DNA methylation level of L1_Md_Tf (L1) obtained by deep hairpin bisulfite sequencing (DHBS) and deep single strand bisulfite sequencing (DSSBS).** DNA methylation analysis of L1 in germ cells and maternal and paternal pronuclei at different timepoints of the developing zygote with DHBS showed the same overall methylation level as the methylation of L1 with DSSBS. (PNG 95 KB)

Additional file 3: **5-ethynyl-2′-deoxyuridine (EdU) incorporation and phosphorylated Histone variant H2A.X (γH2A.X) staining of zygotes inhibited with aphidicolin.** Aphidicolin-treated zygotes (4 to 14 h) did not show any incorporation of nucleotides but still showed expansion of the pronuclei. PB, polar body. (PNG 1 MB)

Additional file 4: **The influence of SAMase expression in zygotes on 5mC.** (A) 5mC immunofluorescence (IF) staining in control and SAMase expressing 14-hour *in vitro* fertilization (IVF) post-replicative zygotes. (B) 5mC IF staining in control and SAMase expressing two-cell embryos. PB, polar body. (PNG 2 MB)

Additional file 5: **Representative pictures of cleavage stage embryos used for hairpin bisulfite analysis of L1Md_Tf (L1), major satellites (mSat) and IAPLTR1 (IAP) from day 2 post-fertilisation (2 dpc, days post-coitum: late 4-cell to early 8-cell stage), 2.5 dpc (early morula: 16 cell stage), 2.5 dpc (late morula stage) to 3.5 dpc (blastocyst stage).** (PNG 1 MB)

Additional file 6: **List of all analyzed samples with number of reads and DNA methylation states.** Samples analyzed with Hairpin bisulfite sequencing with number of reads (#reads), number of analyzed CpGs (#CpGs), conversion rate of the hairpin linker (conversion), and ratio of fully methylated (mC/mC), hemimethylated (C/mC, mC/C), or unmethylated (C/C) CpG positions. (# indicates biological replica). Note that in bisulfite sequencing, unconverted cytosine (C) must be considered as 5-methylcytosine (5mC) or 5-hydroxymethylcytosine (5hmC) and converted C as unmodified C, 5-formylcytosine (5fC), or 5-carboxycytosine (5caC), for example mC = 5mC/5hmC C = C/fC/5caC. (XLSX 17 KB)

## Abbreviations

5mC: 5-methylcytosine
5hmC: 5-hydroxymethylcytosine
5fC: 5-formylcytosine
5caC: 5-carboxylcytosine
DHBS: Deep hairpin bisulfite sequencing
dpc: Days post-coitum
ESCs: embryonic stem cells
hpf: Hours post-fertilization
IAP: intracisternal A-particle-LTR1 ( IAPLTR1)
IF: Immunofluorescence
IVF: *In vitro* fertilization
L1: Line1Md_Tf
mSat: Major satellites
PGCs: Primordial germ cells
PN: Pronuclear stage
SAMase: S-adenosylmethionine hydrolase.
