# Supplementary figures and images for: Selective impairment of methylation maintenance is the major cause of DNA methylation reprogramming in the early embryo
Source: Epigenetics Chromatin. 2015 Jan 9;8:1. doi: 10.1186/1756-8935-8-1 (PMC4304184; doi:10.1186/1756-8935-8-1)

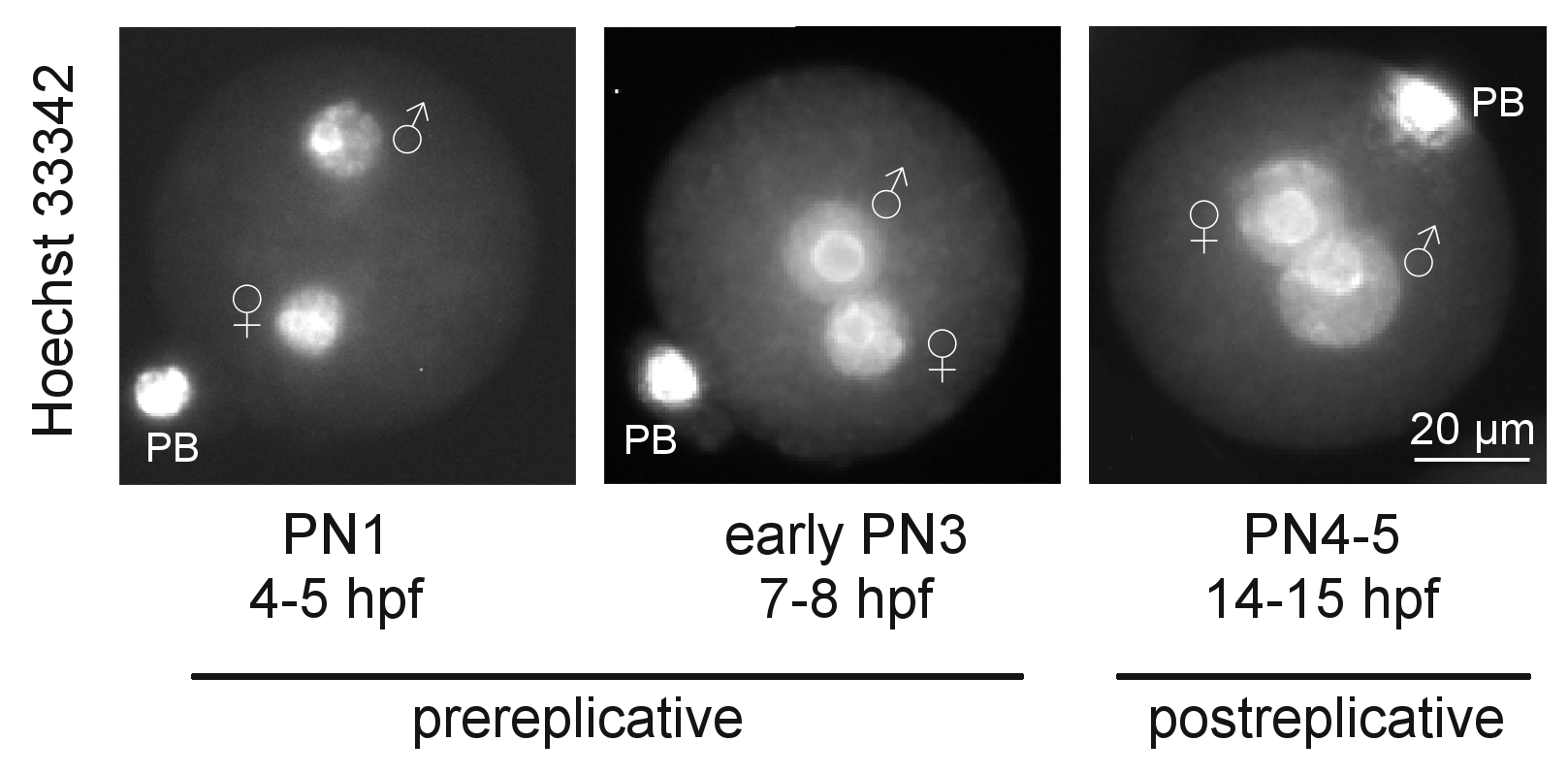

Supplement: Supplementary file 1 — Additional file 1: Representative images of Hoechst 33342-stained mouse zygotes. Discrimination of developed zygotes was performed by hours and the morphology of the pronuclei (PN) as described previously [16]. PN1 and early PN3 represent pre-replicative PN stages and PN4 to PN5 the post-replicative PN stages. PB, polar body. (PNG 269 KB) [file 13072_2014_345_MOESM1_ESM.png]

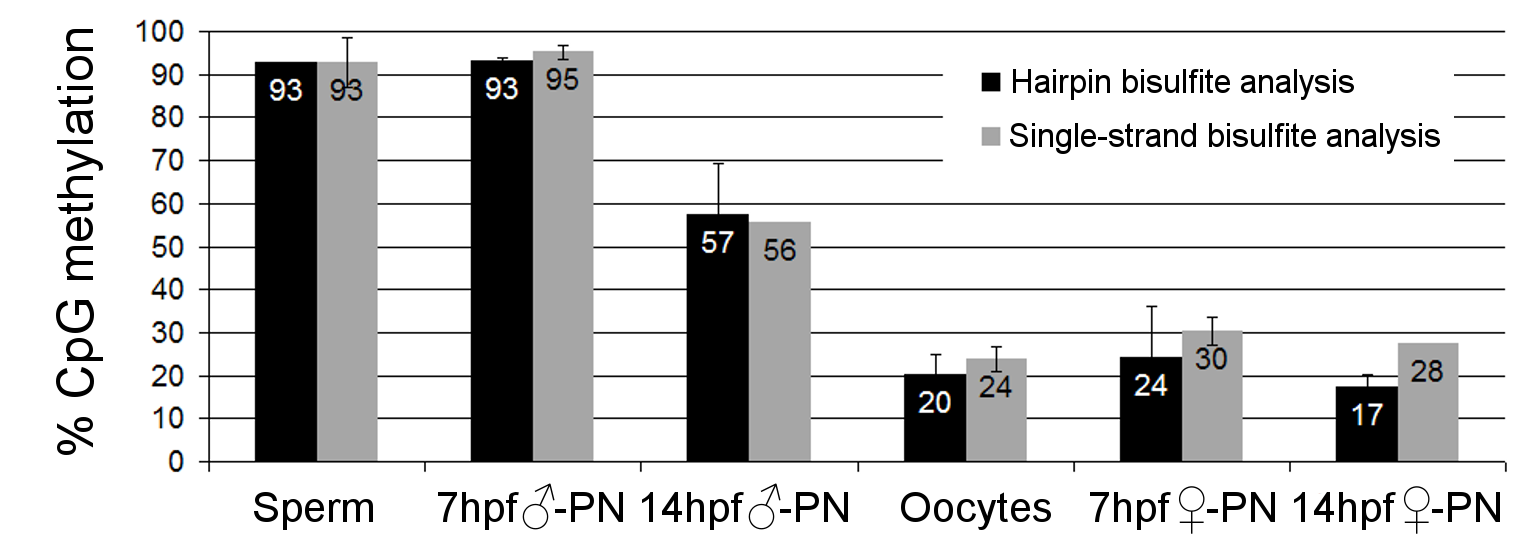

Supplement: Supplementary file 2 — Additional file 2: Comparison of DNA methylation level of L1_Md_Tf (L1) obtained by deep hairpin bisulfite sequencing (DHBS) and deep single strand bisulfite sequencing (DSSBS). DNA methylation analysis of L1 in germ cells and maternal and paternal pronuclei at different timepoints of the developing zygote with DHBS showed the same overall methylation level as the methylation of L1 with DSSBS. (PNG 95 KB) [file 13072_2014_345_MOESM2_ESM.png]

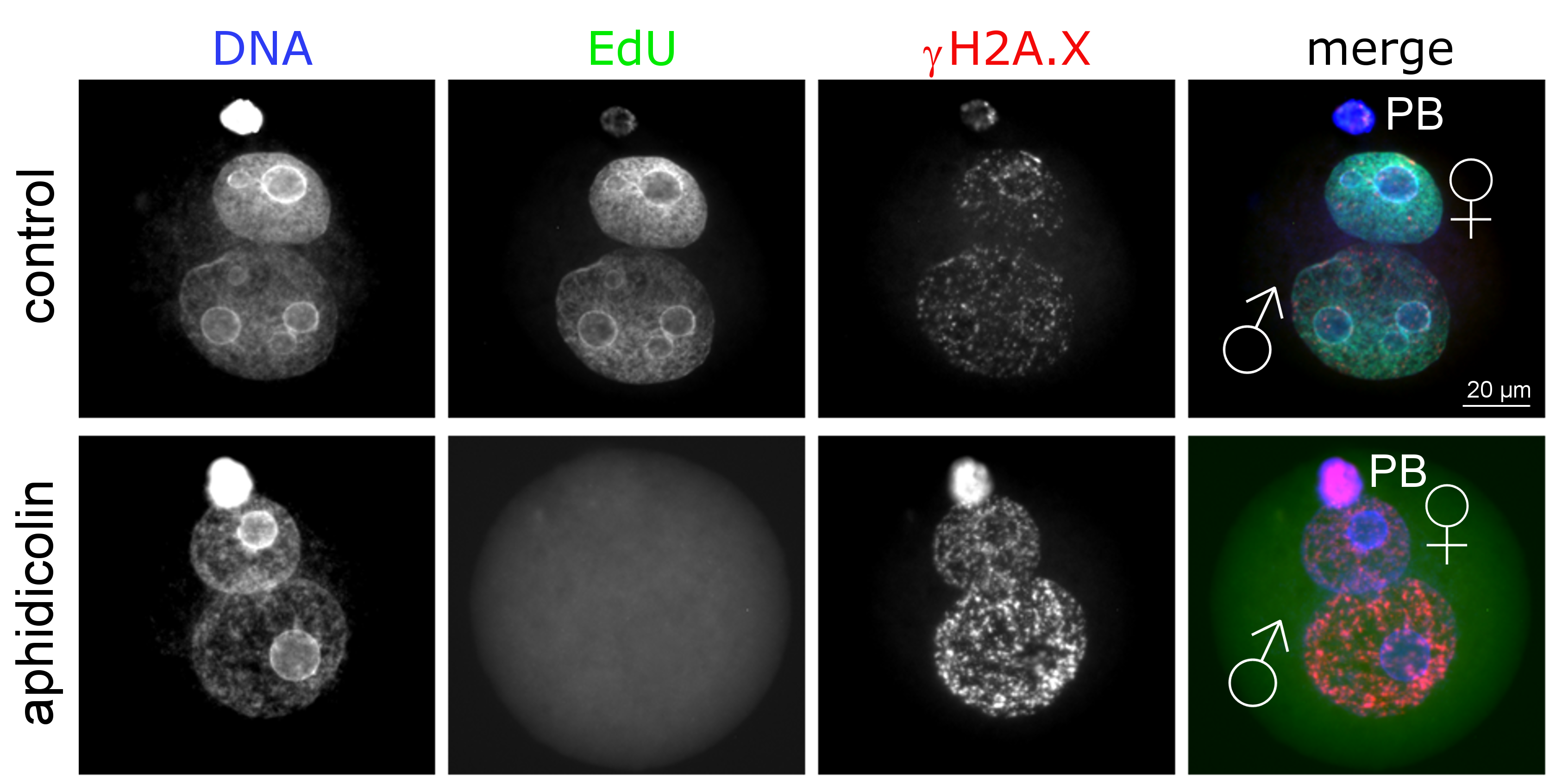

Supplement: Supplementary file 3 — Additional file 3: 5-ethynyl-2′-deoxyuridine (EdU) incorporation and phosphorylated Histone variant H2A.X (γH2A.X) staining of zygotes inhibited with aphidicolin. Aphidicolin-treated zygotes (4 to 14 h) did not show any incorporation of nucleotides but still showed expansion of the pronuclei. PB, polar body. (PNG 1 MB) [file 13072_2014_345_MOESM3_ESM.png]

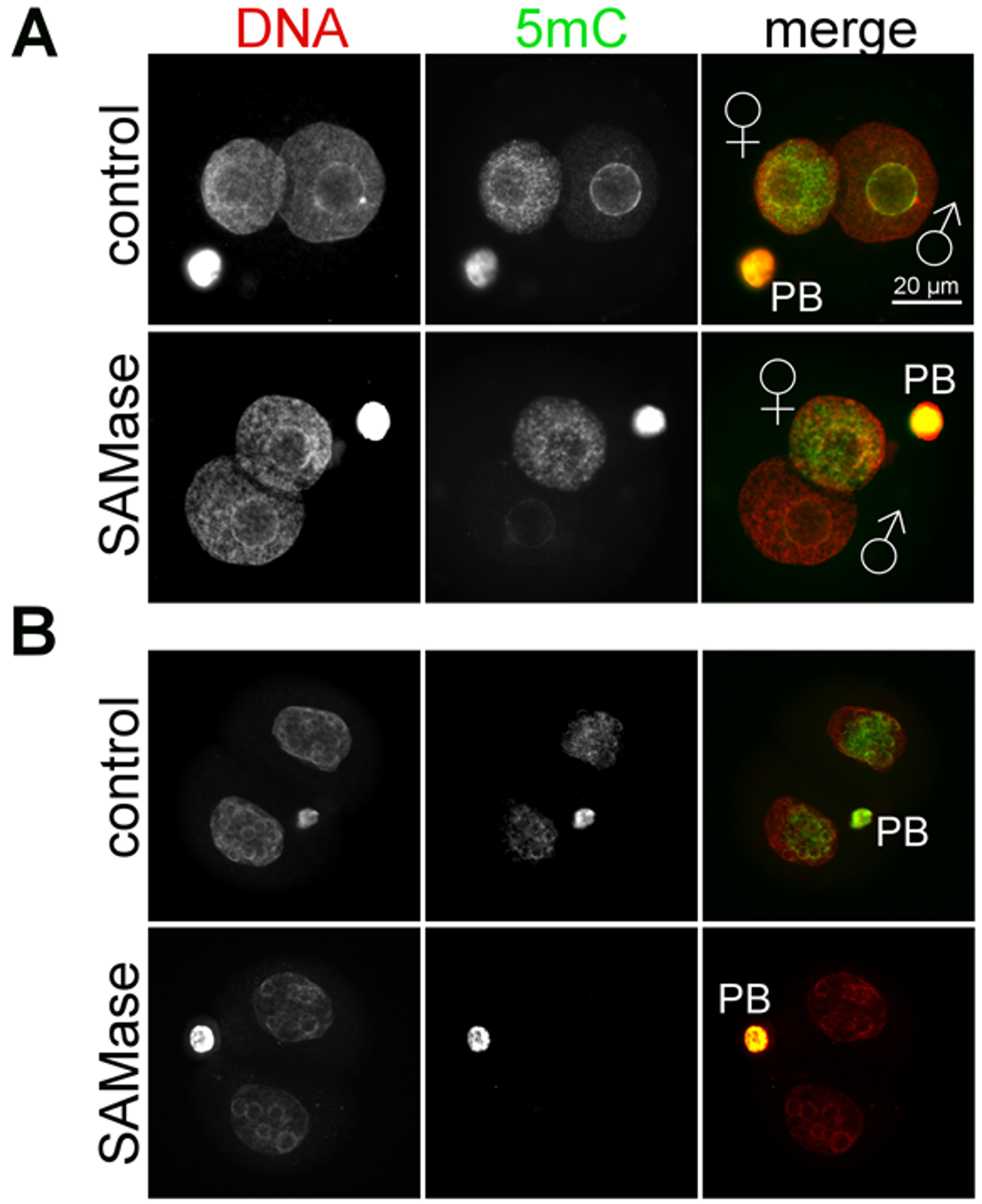

Supplement: Supplementary file 4 — Additional file 4: The influence of SAMase expression in zygotes on 5mC. (A) 5mC immunofluorescence (IF) staining in control and SAMase expressing 14-hour in vitro fertilization (IVF) post-replicative zygotes. (B) 5mC IF staining in control and SAMase expressing two-cell embryos. PB, polar body. (PNG 2 MB) [file 13072_2014_345_MOESM4_ESM.png]

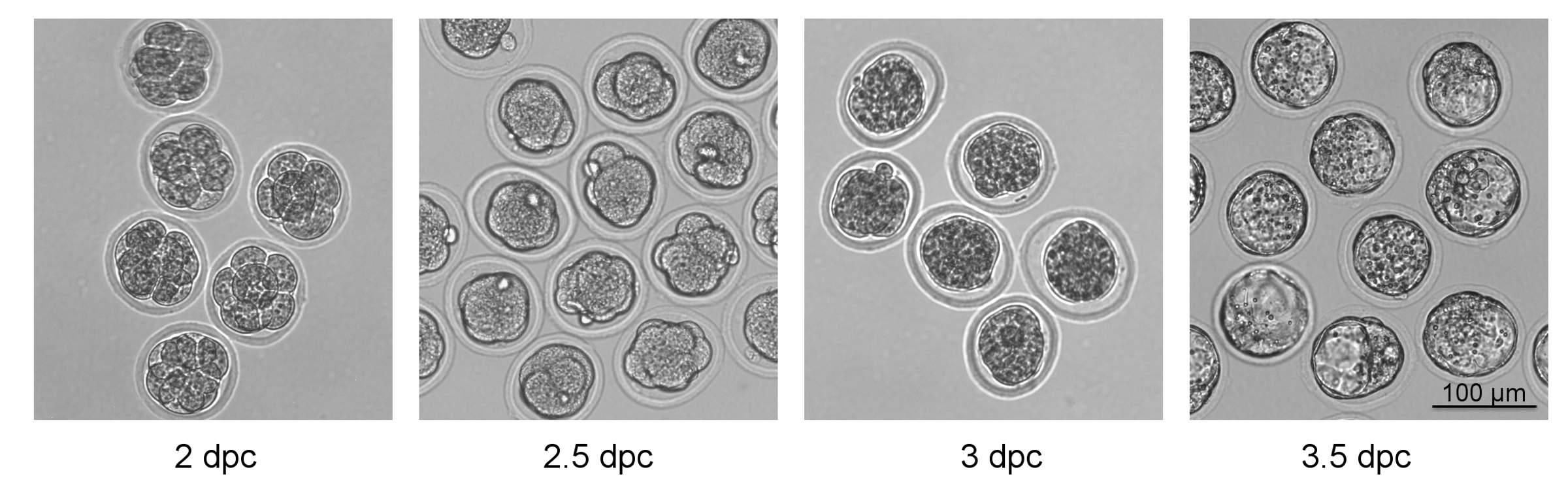

Supplement: Supplementary file 5 — Additional file 5: Representative pictures of cleavage stage embryos used for hairpin bisulfite analysis of L1Md_Tf (L1), major satellites (mSat) and IAPLTR1 (IAP) from day 2 post-fertilisation (2 dpc, days post-coitum: late 4-cell to early 8-cell stage), 2.5 dpc (early morula: 16 cell stage), 2.5 dpc (late morula stage) to 3.5 dpc (blastocyst stage). (PNG 1 MB) [file 13072_2014_345_MOESM5_ESM.png]
